# Population Genomics of Reduced Vancomycin Susceptibility in *Staphylococcus aureus*

**DOI:** 10.1128/mSphere.00094-16

**Published:** 2016-07-20

**Authors:** Lavanya Rishishwar, Colleen S. Kraft, I. King Jordan

**Affiliations:** a School of Biology, Georgia Institute of Technology, Atlanta, Georgia, USA; b Applied Bioinformatics Laboratory, Atlanta, Georgia, USA; c PanAmerican Bioinformatics Institute, Cali, Valle del Cauca, Colombia; d Pathology and Laboratory Medicine, Emory University School of Medicine, Atlanta, Georgia, USA; e Antibiotic Resistance Center, Emory University School of Medicine, Atlanta, Georgia, USA; Centers for Disease Control and Prevention

**Keywords:** *Staphylococcus aureus*, antibiotic resistance, genomics, population genetics, vancomycin

## Abstract

The increased prevalence of vancomycin-intermediate *Staphylococcus aureus* (VISA) is an emerging health care threat. Genome-based comparative methods hold great promise to uncover the genetic basis of the VISA phenotype, which remains obscure. *S. aureus* isolates were collected from a single individual that presented with recurrent staphylococcal bacteremia at three time points, and the isolates showed successively reduced levels of vancomycin susceptibility. A population genomic approach was taken to compare patient *S. aureus* isolates with decreasing vancomycin susceptibility across the three time points. To do this, patient isolates were sequenced to high coverage (~500×), and sequence reads were used to model site-specific allelic variation within and between isolate populations. Population genetic methods were then applied to evaluate the overall levels of variation across the three time points and to identify individual variants that show anomalous levels of allelic change between populations. A successive reduction in the overall levels of population genomic variation was observed across the three time points, consistent with a population bottleneck resulting from antibiotic treatment. Despite this overall reduction in variation, a number of individual mutations were swept to high frequency in the VISA population. These mutations were implicated as potentially involved in the VISA phenotype and interrogated with respect to their functional roles. This approach allowed us to identify a number of mutations previously implicated in VISA along with allelic changes within a novel class of genes, encoding LPXTG motif-containing cell-wall-anchoring proteins, which shed light on a novel mechanistic aspect of vancomycin resistance.

**IMPORTANCE** The emergence and spread of antibiotic resistance among bacterial pathogens are two of the gravest threats to public health facing the world today. We report the development and application of a novel population genomic technique aimed at uncovering the evolutionary dynamics and genetic determinants of antibiotic resistance in *Staphylococcus aureus*. This method was applied to *S. aureus* cultures isolated from a single patient who showed decreased susceptibility to the vancomycin antibiotic over time. Our approach relies on the increased resolution afforded by next-generation genome-sequencing technology, and it allowed us to discover a number of *S. aureus* mutations, in both known and novel gene targets, which appear to have evolved under adaptive pressure to evade vancomycin mechanisms of action. The approach we lay out in this work can be applied to resistance to any number of antibiotics across numerous species of bacterial pathogens.

## Abstract (precis)

The emergence and spread of antibiotic resistance among bacterial pathogens are two of the gravest threats to public health facing the world today. We report the development and application of a novel population genomic technique aimed at uncovering the evolutionary dynamics and genetic determinants of antibiotic resistance in *Staphylococcus aureus*. This method was applied to *S. aureus* cultures isolated from a single patient who showed decreased susceptibility to the vancomycin antibiotic over time. Our approach relies on the increased resolution afforded by next-generation genome-sequencing technology, and it allowed us to discover a number of *S. aureus* mutations, in both known and novel gene targets, which appear to have evolved under adaptive pressure to evade vancomycin mechanisms of action. The approach we lay out in this work can be applied to resistance to any number of antibiotics across numerous species of bacterial pathogens.

## INTRODUCTION

*Staphylococcus aureus* infections are a major cause of mortality and morbidity worldwide (1). Incidence rates range from 20 to 50 cases per 100,000 individuals with 10 to 30% mortality (2, 3). Elimination of *S. aureus* infections typically requires a prolonged course of antibiotics (4), and evolved antibiotic resistance is a major challenge to the effective treatment of *S. aureus* infections (5). *S. aureus* isolates resistant to the methicillin antibiotic were first identified in the early 1960s (6, 7). Since that time, methicillin-resistant *S. aureus* (MRSA) has become increasingly widespread and is now a leading cause of hospital-acquired infections (8). The mechanism of methicillin resistance is well understood and involves the acquisition of a single-mobile-element-borne gene *mecA* (9, 10).

MRSA is so common that hospital-acquired infections are typically assumed to be methicillin resistant, and patient treatment is accordingly initiated with vancomycin, which has emerged as the mainstay of *S. aureus* infection therapy (11). Despite the overall efficacy of vancomycin as an antibiotic, *S. aureus* resistance to vancomycin is becoming increasingly prevalent (12, 13). The first cases of intermediate resistance to vancomycin were identified in 1996 (14), and fully resistant strains were later found in 2002 (15). As is the case for methicillin resistance, full vancomycin resistance is based on the acquisition of a single gene, *vanA* (16, 17). Vancomycin-resistant *S. aureus* (VRSA) is extremely rare, with only 12 cases reported in the United States since 2002 (15), and therefore does not represent an urgent public health threat.

Vancomycin-intermediate resistance is defined on the basis of the range of MICs of the antibiotic needed to inhibit growth. *S. aureus* strains that show MICs from 3 to 8 µg/ml are characterized as vancomycin-intermediate *S. aureus* (VISA) (13). Unlike VRSA, the incidence of VISA infections is steadily rising (12, 13), a phenomenon referred to as vancomycin MIC creep (18), and VISA therefore does pose a serious potential threat to the effective antibiotic treatment of *S. aureus*.

At this time, the precise genetic basis of the VISA phenotype is unknown. VISA does not appear to be a single gene phenomenon, as seen for MRSA and VRSA, and different VISA cases may be caused by different gene sets (19). The lack of a clear-cut gene-to-VISA relationship, combined with the public health relevance of increased vancomycin resistance, has stimulated numerous attempts to uncover the genetic basis of VISA (20–26). One specific approach that can be taken to address this question entails the comparison of genome sequences between *S. aureus* isolates with different vancomycin susceptibility profiles (22, 27–33). The goal of such studies is to identify mutated genes that are (i) exclusively found in isolates with reduced vancomycin susceptibility and (ii) encode proteins with plausible roles in the VISA phenotype (e.g., cell-wall-related functions).

The genomic approach to studying VISA is typically performed by comparing genome consensus sequences that are assembled from overlapping sequence reads that cover the entire *S. aureus* genome multiple times. The consensus sequence approach implicitly assumes that patient isolates are monoclonal, since it collapses all observed site-specific variation among sequence reads to a single consensus sequence, i.e., it considers sequence variation among colocated reads as noise that should be removed. Nevertheless, as the phenomenon of vancomycin creep is based on evolution, it is highly likely that many *S. aureus* patient isolates are in fact polyclonal and characterized by subpopulations that bear VISA-related mutations at low frequencies. In fact, it is known that there are many cases of heterogeneous VISA (hVISA) that correspond precisely to this description (34). hVISA isolates have overall MICs that fall within the vancomycin-susceptible range, but they also contain low-frequency subpopulations that are demonstrably less susceptible (35). For such hVISA populations, treatment with vancomycin could cause less-susceptible subpopulations to increase in frequency along with their relevant VISA-related mutations (36).

**FIG 1:**
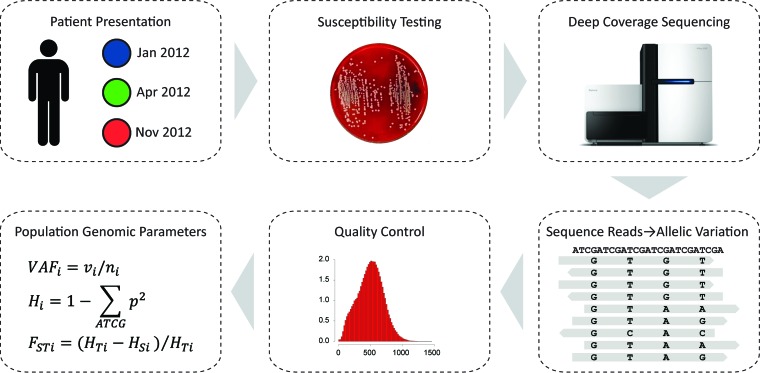
Overview of the *S. aureus* population genomic approach used in this study.

In this study, a novel population genomic approach was developed and applied in an effort to characterize the genomic determinants of the emergence of reduced vancomycin susceptibility in a single patient (Fig. 1). This patient was admitted to Emory Healthcare with recurrent *S. aureus* infections several times over the course of 1 year, and at each successive time point, the patient’s isolates showed increased vancomycin MIC levels. The population genomic approach employed here does not assume that the patient’s *S. aureus* isolates are monoclonal. Instead, it uses site-specific *S. aureus* sequence variation uncovered by deep sequencing to model the allele frequency dynamics between patient isolates that were taken from different time points and showed different vancomycin MICs. This approach provides additional resolution for the study of the evolution of vancomycin resistance and for the detection of VISA-implicated mutations compared to the consensus sequence approach. A number of mutations in candidate genes previously implicated in the VISA phenotype were found using this approach along with mutations distributed among members of a novel class of VISA-related genes that encode cell-wall-related functions.

## RESULTS AND DISCUSSION

### Presenting patient.

In January 2012, a 50-year-old African-American male with a history of uncontrolled diabetes and end-stage renal disease requiring hemodialysis presented at Emory Healthcare and was found to have an *S. aureus* bloodstream infection. The patient was treated with intravenous vancomycin for 22 days for this bacteremia. The patient’s *S. aureus* isolate from this time point was determined to be methicillin resistant and vancomycin susceptible with an MIC of 1.5 µg/ml by Etest on Mueller-Hinton agar. The patient was discharged and readmitted in April with recurrent *S. aureus* bloodstream infection, complicated by septic arthritis of the shoulder. Vancomycin treatment was reinitiated after the infection was cultured and continued for 44 days. The patient’s second time point isolate was found to have a vancomycin MIC of 2.0 µg/ml. The patient returned in August with recurrent *S. aureus* infection and received treatment with 40 additional days of intravenous vancomycin treatment. In November, the patient was again readmitted with recurrent *S. aureus* bloodstream infection, complicated by discitis and osteomyelitis of his T7 and T8 vertebral bodies. The patient’s isolate from this time point was found to have a vancomycin MIC of 3.0 µg/ml, initially by automated susceptibility testing, and then determined by Etest on Mueller-Hinton agar, which is considered to be at the lower end of the VISA MIC range (13). The patient was therefore treated with daptomycin after this bacteremia. Patient isolates from three time points (January, April, and November; Table 1) were preserved and subsequently treated for DNA extraction (see Materials and Methods).

**TABLE 1:** Patient’s *S. aureus* isolate sites, phenotypes, and genome sequences

Isolate	Isolation^a^	Total no. of bases sequenced	Coverage (fold)	Patient diagnosis	Isolate site	Vancomycin MIC (µg/ml)	Class	SRA accession no.
VSSA-T1	Jan 2012	1,494,782,530	514.27	Bacteremia	Blood	1.5	VSSA	SRX689719
VSSA-T2	Apr 2012	1,343,767,861	462.32	Bacteremia	Blood	2.0	VSSA	SRX689725
VISA-T3	Nov 2012	1,354,762,500	466.10	Bacteremia	Blood	3.0	VISA	SRX689726

Avg		1,397,770,964	480.89					

a The patient’s isolates from three different time points (January [Jan], April [Apr], and November [Nov]) were characterized.

### Isolate genome sequences and phylogeny.

Patient *S. aureus* isolate genome sequences from the three time points were sequenced to ~500× coverage as described in Materials and Methods (Table 1). *De novo* assembly was performed in order to yield sets of consensus sequence contigs for each of the three sets of sequence reads. Sequences of the resulting three unfinished assemblies were compared against all complete *S. aureus* genome sequences available in the NCBI RefSeq database, along with a set of previously published *S. aureus* genome sequences from clinical isolates taken at the same Emory Healthcare laboratory (33), to yield all-against-all pairwise nucleotide distances. Phylogenetic analysis of these distance data was used to choose the closest complete *S. aureus* genome sequence for subsequent read-to-genome mapping and single nucleotide polymorphism (SNP) calling.

**FIG 2:**
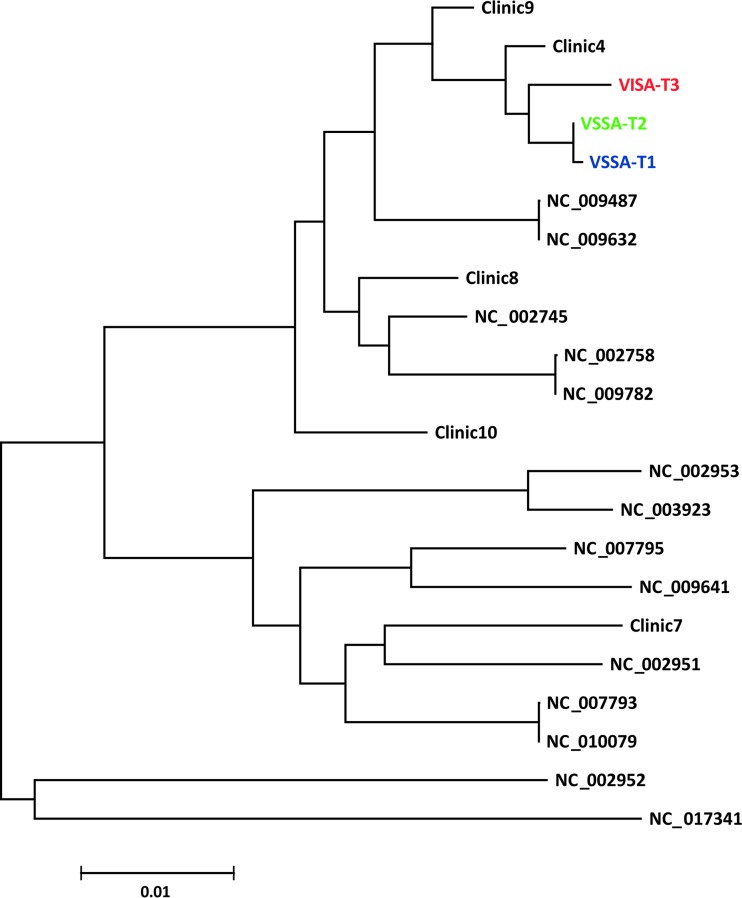
Phylogeny of *S. aureus* isolates based on genome-wide average nucleotide distances (scale bar). Patient isolates from three time points are shown as VSSA-T1, VSSA-T2, and VISA-T3.

*S. aureus* genome sequences from the patient isolates (VSSA-T1, VSSA-T2, and VISA-T3 where T1, T2, and T3 stand for time points 1, 2, and 3) form a single monophyletic cluster and are most closely related to two of the previously characterized clinical isolates (Fig. 2). The most closely related complete *S. aureus* reference sequences correspond to a pair of genomes from a previous study (NCBI RefSeq accession no. NC_009487 and NC_009632), which also documented and traced the emergence of VISA over time in a single patient (32). The tight phylogenetic clustering seen for the three time point isolates characterized here is consistent with a single infection followed by *in situ* evolution of the patient’s strains leading to reduced vancomycin susceptibility, as opposed to multiple infections with distinct *S. aureus* strains having different vancomycin susceptibilities. The phylogenetic analysis does however show substantial variation among the patient’s three time point sequences, far more than seen for the two strains from the previous study, which were collected 3 months apart in time. Furthermore, the VISA isolate sequence from time point three diverges markedly compared to the sequences from the first two time points. This suggests the possibility of a particularly rapid period of evolution, after an initial slower phase, leading to the VISA phenotype at the third time point. However, it likely also reflects, to some extent, the fact that there are only 3 months between time points one and two compared to 7 months between time points two and three.

### Overall reduction in variation.

Having defined the closest complete *S. aureus* reference sequences to the patient’s three isolate sequences, individual reads for each time point isolate were mapped to the reference sequences. As the two reference sequences are highly similar (>99.99% identity), all results based on the read-to-genome mapping were qualitatively identical for each reference sequence. Here, results for the NC_009487 reference sequence are reported.

**FIG 3:**
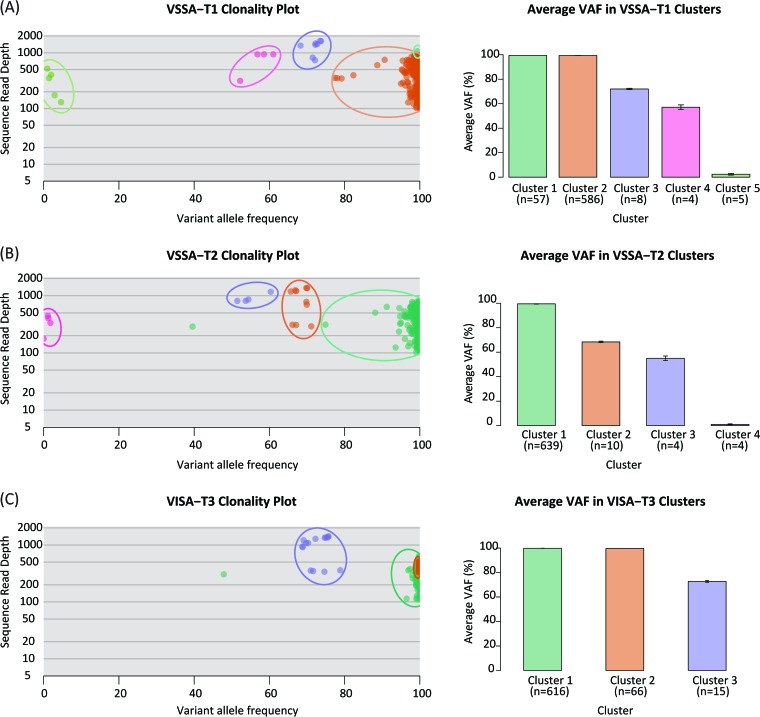
Clonality of *S. aureus* patient isolates from three time points. Clusters of variants with similar variant allele frequencies (VAFs) characterize individual clones. The number of clones, along with their average VAFs, are shown for each time point isolate.

The high sequencing depth achieved for each time point isolate (Table 1) provided substantial resolution to search for genome sequence variation within individual isolate samples. In other words, individual patient isolates could be evaluated to assess their clonality or lack thereof. To do this, a technique developed to evaluate the clonality of cancer samples was applied to the isolate genome sequence reads (37). This approach treats individual reads and their site-specific base calls as alleles in a population. The variant allele frequencies, i.e., the frequency of alleles that differ from the reference sequence, are compared to the sequencing depth in order to identify colocated clusters that are likely to correspond to individual clones within a sample of cells. Applying this technique to the patient’s time point genome sequence reads revealed that the patient isolates are polyclonal and that there was a monotonic reduction in the total number of clones from time point one to time point three (Fig. 3). This is consistent with an overall reduction of variation over time and can be attributed to the bottleneck that bacterial populations experience when exposed to antibiotic treatment.

**FIG 4:**
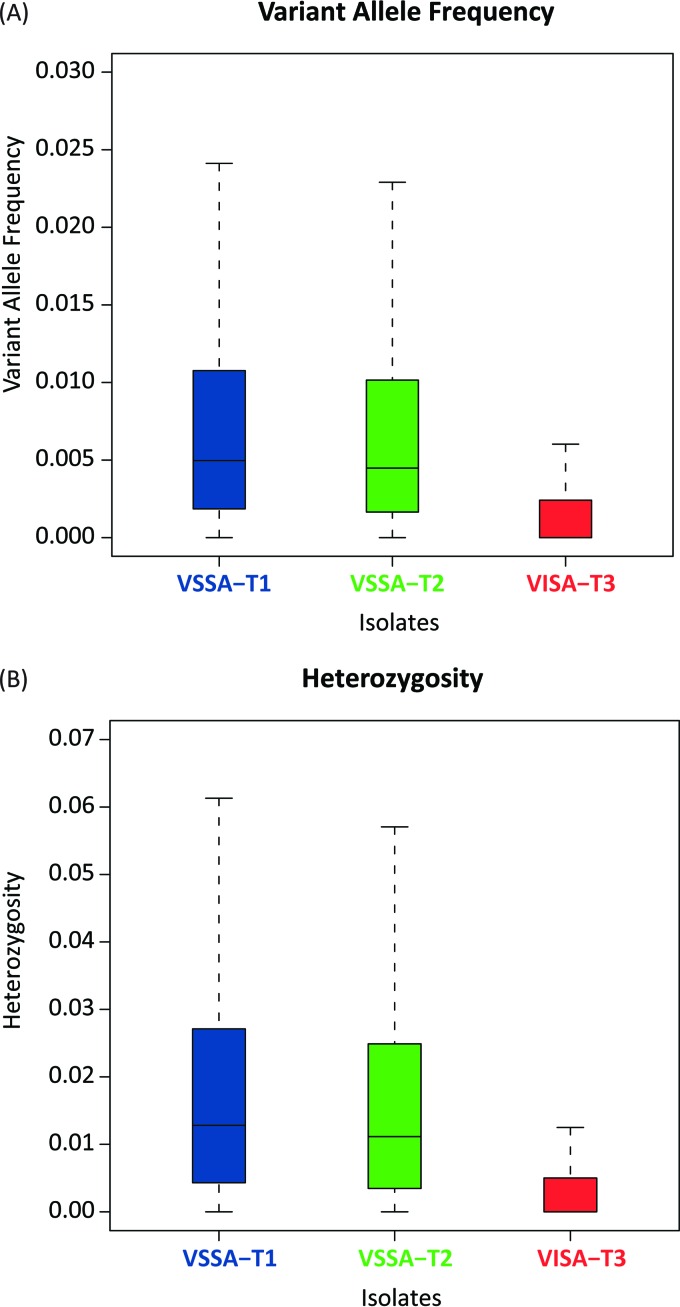
Levels of *S. aureus* genome variation seen for patient isolates from three time points. Box plots are shown for site-specific distributions of variant allele frequency and heterozygosity. For a given mapped position in a time point isolate, the variant allele frequency is defined as the number of nonreference bases divided by the total number of mapped bases. Heterozygosity is a measure of allelic diversity at each of the mapped positions (see Materials and Methods).

The overall levels of variation within each time point’s isolate population were further quantified using distributions of both the site-specific variant allele frequencies and site-specific heterozygosity values (see Materials and Methods). Both of these metrics reveal a monotonic reduction in the overall sequence variation across the three time points, consistent with the results from the clonality analysis (Fig. 4). There is a particularly marked reduction in variation seen for the VISA isolate from time point three. Differences in the levels of variation between the time points are all highly significant (*P* ≈ 0 by Mann-Whitney U test).

### Allele frequency spectrum shift.

**FIG 5:**
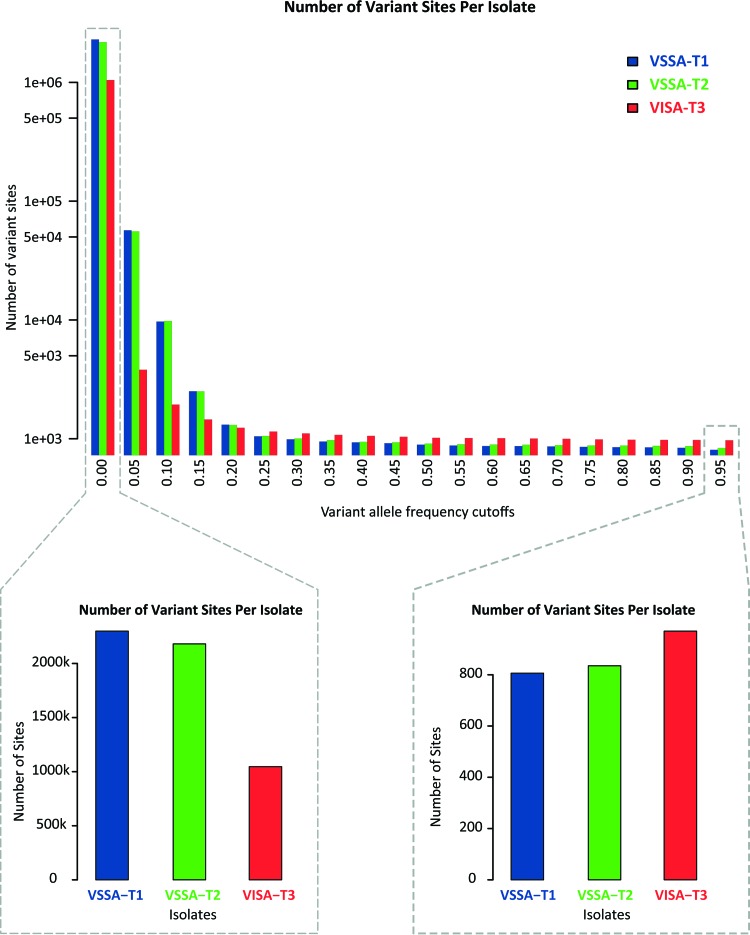
*S. aureus* variant allele count distribution for patient isolates from three time points. Variant allele counts are shown across a range of variant allele frequency cutoffs (top panel). Insets are shown for the two most extreme cutoff values (bottom panel).

The allele frequency spectra for the patient *S. aureus* isolate sequences were characterized in order to further interrogate the population evolutionary dynamics that accompanied the decrease in vancomycin susceptibility over time. To do this, variant allele counts for the three time points were calculated across an increasingly stringent series of variant allele frequency cutoff levels (Fig. 5). At low cutoff levels, all or most of the variant sites are considered, whereas at higher cutoff levels, only variant alleles that are found at high frequency are counted. At the 0 cutoff level, which is equivalent to Fig. 4B, all variant alleles are considered. At this level, and for other low cutoff levels (0 to 0.2), time point one shows the most variant alleles, followed closely by time point two; then, there is a precipitous drop in the number of variant alleles at time point three. This is consistent with the overall reduction in variation across time points documented in the previous section. As the variant allele frequency cutoff increases, this pattern begins to shift. At higher cutoff levels, i.e., when only high-frequency variants are counted (>0.25), time point three shows the highest number of variants, followed by time points two and one, respectively (Fig. 5). This result indicates that, despite the overall reduction in variation over time that was caused by antibiotic treatment, a number of low-frequency variants were swept to high frequency levels (or fixed) in the VISA time point population.

These kinds of VISA-characteristic mutations are likely to point to genomic loci (genes) that are implicated in the emergence of the VISA phenotype. Furthermore, the overall reduction in variation over time, i.e., the population bottleneck, provides a background against which VISA site-specific allele frequency shifts can be parameterized to look for VISA-characteristic mutations that are statistically significant outliers. Two statistical tests based on this logic were applied to the time point-specific allelic variant data to look for sites that are most likely to be related to the emergence of VISA in this patient. The first test is based on the distribution of site-specific variant allele frequency differences, and the second test is based on the distribution of site-specific population differentiation levels (polarized *F_ST_*). All ~2 million genomic positions are considered in these tests, yielding high statistical power. These tests are further described in Materials and Methods.

**FIG 6:**
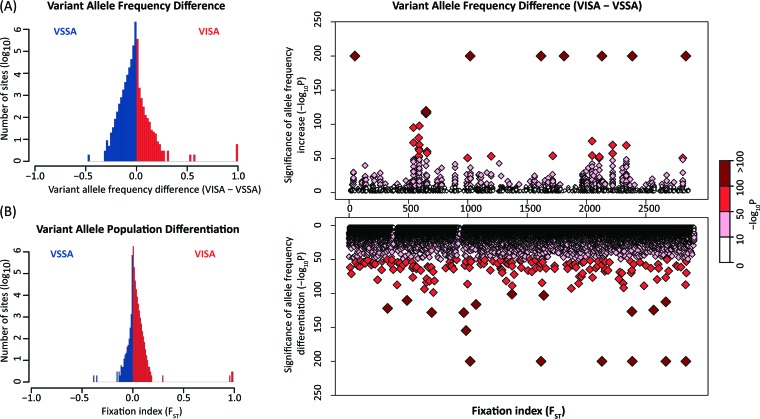
Site-specific values of *S. aureus* variant allele frequency differences and variant allele population differentiation between VSSA and VISA patient isolates. (Left) Distributions showing site-specific values of variant allele frequency change and population differentiation (*F_ST_*). Site-specific values are colored according to whether they are greater in VSSA (blue) or VISA (red). (Right) Manhattan plots showing the corresponding statistical significance levels for site-specific changes in variant allele frequencies and variant allele population differentiation in the VISA isolate.

The variant allele frequency difference distribution and the *F_ST_* were computed for the vancomycin-susceptible *S*. *aureus* isolate at time point one (VSSA-T1) versus the VISA time point and for the VSSA-T2 versus VISA time points. The results are qualitatively similar, and results of the comparisons between the VSSA-T1 and VISA time points are reported here (Fig. 6). The overall variant allele frequency difference distribution is shifted toward VSSA, consistent with the overall reduction in variation over time. However, the VISA-specific allele frequency differences have a broader distribution with a long tail, consistent with the allele frequency distribution shift previously described. The statistical significance values of the site-specific allele frequency differences were used to rank VISA-implicated mutations for subsequent interrogation (Fig. 6).

The VSSA-to-VISA *F_ST_* distribution shows a different pattern with the majority of site-specific polarized *F_ST_* values being VISA related. Nevertheless, the VISA side of the distribution is broader and shows a longer tail as seen for the variant allele frequency difference distribution. The statistical significance values of the site-specific polarized *F_ST_* values were also used to rank VISA-implicated mutations for subsequent interrogation (Fig. 6).

### Candidate gene VISA mutations.

The utility of the population genomic approach employed here for the detection of VISA-related mutations was evaluated by searching for statistically significant mutations in candidate genes that had previously been implicated in the VISA phenotype. To do this, a database of 24 VISA-implicated candidate genes based on the results of 17 previous studies (reviewed in reference 19) was created. Of these 24 genes, 12 bear statistically significant nonsynonymous mutations (Table 2; see Table S1 in the supplemental material); furthermore, the total number of significant mutations found in the candidate genes is higher than the expected number based on the distribution of significant mutations across the entire genome (*P* = 8.1 × 10^−9^ by Fisher exact test). A similar approach was used to interrogate a larger set of 237 genes, which had previously been implicated in the VISA phenotype by virtue of their differential expression in VISA compared to related VSSA strains (26). Of the 237 genes, 145 bear statistically significant nonsynonymous mutations, and there is also a significant increase in the total number of significant mutations that map to these genes (*P* = 2.8 × 10^−3^ by Fisher exact test). These results serve as a proof of principle for the population genomic approach employed here and underscore the power of the statistical tests employed.

10.1128/mSphere.00094-16.1 Table S1  List of previously reported vancomycin resistance mutations detected in the isolates analyzed here. For each mutation listed, the genomic position in the reference genome NC_009487, the nucleotide change, the NCBI identifier (ID), the gene name, the function of the gene that the mutation is present in, the amino acid change, and the Grantham score of the effect of mutation are provided. Download Table S1, XLSX file, 0.01 MB. Copyright © 2016 Rishishwar et al. 2016 Rishishwar et al. This content is distributed under the terms of the Creative Commons Attribution 4.0 International license.

**TABLE 2:** Genes reported in the literature to be implicated in the VISA phenotype

Gene	Protein function
*fmtC*	Protein of unknown function DUF470
*graR*	Two-component transcriptional regulator, winged helix family
*graS*	Integral membrane sensor signal transduction histidine kinase
*isdE*	Periplasmic binding protein
*prsA*	PpiC-type peptidyl-prolyl *cis*-*trans* isomerase
*spoVG*	SpoVG family protein
*trfA*	Negative regulator of genetic competence
*vraF*	ABC transporter related
*vraG*	Hypothetical protein
*yycF*	Two-component transcriptional regulator, LuxR family
*yycG*	Integral membrane sensor signal transduction histidine kinase
*yycH*	Two-component transcriptional regulator, winged helix family

### Novel gene VISA mutations.

Having established the relevance of the statistically significant VISA-related mutations by virtue of their overabundance in previously identified VISA candidate genes, the presence of such mutations in novel genes not previously implicated in the VISA phenotype was interrogated for clues as to potentially novel mechanisms that underlie the acquisition of VISA in this patient. To do this, mutations were ranked by their statistical significance, and functional enrichment analysis was performed on genes corresponding to the top 500 SNPs. This was done independently for each test, variant allele frequency difference and *F_ST_*, and for both tests combined.

**FIG 7:**
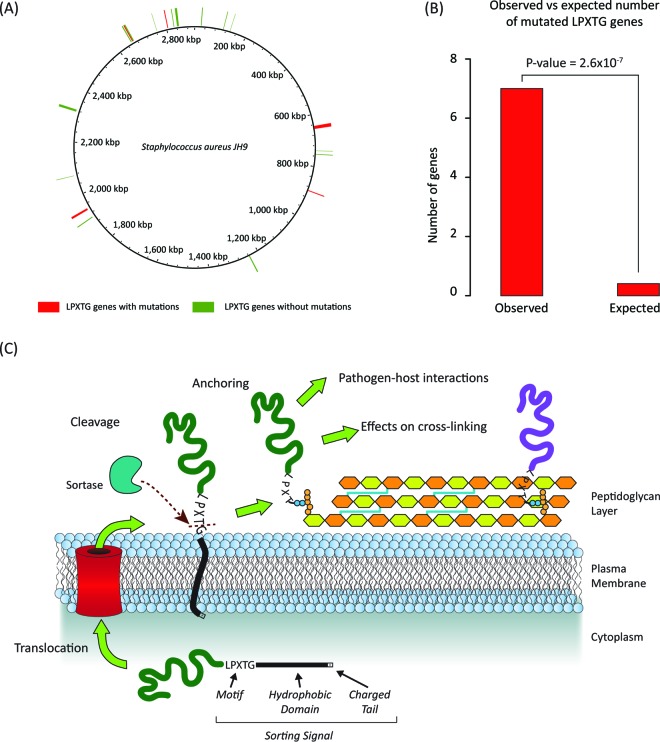
The LPXTG cell wall anchor domain protein family. (A) *S. aureus* genomic distribution of LPXTG motif-containing protein-coding genes. (B) Observed versus expected numbers of LPXTG motif-containing protein-coding genes that overlap with the top 500 statistically significant mutations (Fig. 6). (C) Schematic representation of cell-wall-related LPXTG motif-containing protein functions.

One particularly interesting set of genes, which turned up as enriched in all three instances of this approach, encodes the LPXTG cell wall anchor domain protein family. Genes encoding 7 out of 20 members of this family contain statistically significant mutations, far more than is expected by chance (Fig. 7A and B). Members of this family are surface proteins that perform a variety of functions related to pathogen-host interactions, including antigen presentation, induction of platelet aggregation, and maintenance of cell wall integrity (38–40). These proteins all contain an LPXTG sequence motif at their C terminus, which is a cleavage signal that leads to the covalent binding of the proteins to the cell wall (39, 40) (Fig. 7C). The presence of covalently bound LPXTG proteins has been shown to contribute to the regular cross-linked structure of the cell wall peptidoglycan layer (41).

**FIG 8:**
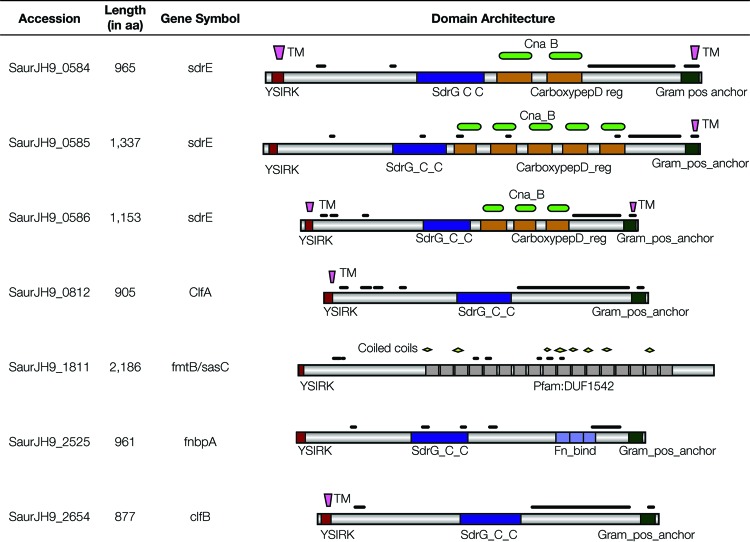
Domain architectures of LPXTG cell wall anchor domain protein family members encoded by genes with VISA-implicated mutations. The NCBI accession number, length (in amino acids [aa]), gene symbol, and domain architecture of each protein are shown. The domains are color coded and shown along with their names. The transmembrane domains (TM), carboxypeptidase regulatory-like (CarboxypepD reg), Gram-positive anchor (Gram_pos_anchor), and the other domain names are described in the text. Black lines above each protein diagram correspond to low-complexity regions.

We evaluated the domain architectures of the seven LPXTG proteins implicated by the three statistical tests in order to better understand the functional characteristics that are shared among them (Fig. 8). Each protein can be seen to contain the YSIRK type signal peptide at the N-terminal domain, followed by low-complexity regions (black lines) and, in most cases, an SdrG glycoprotein adhesion domain. Some of the LPXTG proteins contain additional domains such as carboxypeptidase regulatory-like and/or Cna type B domains. The proteins typically end with another low-complexity region followed by a Gram-positive anchor (shown at the right of the diagram) at the C terminus, which contains the characteristic LPXTG domain. Transmembrane segments are found to be colocated with N- and C-terminal domains. All of these domains point to a functional role for these proteins related to the peptidoglycan cell wall layer.

Mutations to members of this family, such as those seen in the time point three isolate population for this patient, could function to reduce the cross-linking of the peptidoglycan cell wall layer. This is relevant to the VISA phenotype, since the extent to which vancomycin can permeate the *S. aureus* cell wall has previously been shown to be related to the integrity of peptidoglycan cross-linking (42). Specifically, aberrations in the cross-linked peptidoglycan structure could provide false binding sites for vancomycin, thereby reducing its permeation across the cell wall layer. In other words, mutations to members of the LPXTG cell wall anchor domain protein family may result in a lowering of the effective concentration of vancomycin and accordingly reduced susceptibility.

Previous results showing a relationship between the VISA phenotype and an overall reduction in the expression of surface proteins (43) points to another potential mechanism by which mutations to LPXTG cell wall anchor domain proteins could yield reduced vancomycin susceptibility. Since LPXTG motifs are responsible for mediating the proper translocation and anchoring of surface proteins to the peptidoglycan layer, mutations to these proteins could lead to lower overall levels of surface proteins and thus be functionally analogous to the previously observed lower levels of surface protein expression in VISA strains.

### A case of heterogeneous VISA.

The results of the population genomic comparison between VSSA and VISA isolates from the same patient reveal this to be a likely case of heterogeneous VISA (hVISA). hVISA isolates are polyclonal populations wherein the entire isolate population shows a vancomycin MIC within the susceptible range (<2 µg/ml) but a small proportion of clones from the same isolate show an MIC in the intermediate range (3 to 8 µg/ml) (35). In the case of the patient case studied here, the entire isolate population from time point three shows an MIC at the low end of the intermediate range (3 µg/ml). Nevertheless, there are likely to be a number of clones for this same time point that have higher MIC values. This is supported by the fact that most of the statistically significant mutations detected by the population genomic tests employed here have been swept to relatively high frequency but have not been fixed. Furthermore, the patterns of allelic change observed here indicate that there are likely to be clones from the time point one and time point two VSSA isolates, characterized by relatively low-frequency mutations, which also have MICs in the intermediate range.

hVISA is typically detected using a culture-based test referred to as a vancomycin population analysis profile (PAP) (44). This approach is both labor-intensive and time intensive, since it requires a series of continuous MIC readings at different concentrations over 2 to 3 days. The results here point to the possibility of using a population genomic approach, which is made possible by the increasing throughput and decreasing costs of next-generation sequencing techniques, for the characterization of hVISA. Adaptation and standardization of such a genome sequence-based approach to the detection of hVISA could be particularly relevant, given the fact that the retrospective studies have revealed 50% of VSSA isolates to represent cases of hVISA (34). The utility of the population genomic approach is discussed further below.

### Utility of the population genomic approach.

A novel population genomic approach was taken for this study on the genomic basis of the emergence of the VISA phenotype in a single patient over time. Typically, studies of this kind have relied on the comparison of pairs of single genome sequences, each corresponding to a particular level of vancomycin susceptibility, which are in reality consensus sequences generated from the merging of numerous sequence reads covering each position of the genome. This approach assumes that patient isolates are monoclonal, and as such, any observed site-specific variation is considered to be noise that is removed in the process of generating a single genomic consensus sequence.

The population genomic approach employed here relies on deep genomic sequencing that yields high sequence read coverage across the genome (Fig. 1 and Table 1). This high coverage can be used to detect bona fide sequence variation at specific sites that may indicate the presence of more than one clone in a patient isolate. Site-specific variations uncovered by this approach can be considered allelic differences between multiple clones within time points and can also be used to model the population genome dynamics of acquired antibiotic resistance over time. Much of the power of this approach rests on the fact that it considers site-specific variation levels genome-wide, yielding ~2 million data points, which can be used to create distributions of variation that can parameterize statistical tests as shown here (Fig. 6).

Comparison of results based on the more-traditional consensus sequence-based approach with the novel tests employed here underscores the power of the population genomic approach and the extent to which distinct statistical tests are complementary. The genome consensus sequence approach reveals only 12 differences between the VSSA isolate from time point one and the VISA isolate from time point three, whereas the variant allele frequency difference and *F_ST_* tests yield numerous statistically significant results. The large number of significant results returned by these tests could be taken to suggest that it is overly sensitive, but there is a simple solution to this potential problem that was used here, which is known as the outlier approach. In the outlier approach, mutations are ranked accordingly to their statistical significance, and only the most significant mutations are considered. In addition to increasing the stringency of the statistical tests employed, this approach also allows for changing of the threshold for consideration in order to assess the robustness of any functional enrichment analysis performed on the results.

## MATERIALS AND METHODS

### Patient isolates and susceptibility testing.

Patient bloodstream samples at three time points (Table 1) were collected and cultured in the Emory Healthcare microbiology laboratory (under Institutional Review Board approval 50685). For clinical care of the patient, the *S. aureus* isolates were subjected to automated susceptibility testing using the MicroScan WalkAway 96 Plus system (Siemens Healthcare Diagnostics Inc., Tarrytown, NY), and vancomycin susceptibility was confirmed by Etest (bioMérieux, Inc., Durham, NC). The initial subculture plate was entirely swept using a plastic loop (after at least 48 h of incubation) and frozen at −80°C for DNA isolation in order to ensure the representation of isolate subpopulations for subsequent deep sequencing and analysis.

### DNA isolation and genome sequencing.

Each time point isolate colony set was incubated with 50 mg/ml lysozyme and 5 mg/ml lysostaphin at 37°C for 1 h prior to DNA extraction. DNA extraction was performed with the EZ1 Advanced XL system using the DNA Tissue kit and the Bacteria Card (Qiagen, Valencia, CA). Time point isolate DNA sequencing was performed on the Illumina MiSeq instrument using the paired-end read protocol. This resulted in high-coverage (~500×) draft genome sequences used for subsequent analyses.

### Genome sequence analysis.

Genome sequence reads were evaluated for quality using the FastQC program (45). The mean Phred score for all reads was 35, and a Phred cutoff score of 20 was used for removal of low-quality reads and read trimming. Removal of low-quality reads and read trimming were done with the FASTX-toolkit (46). *De novo* genome assemblies for the three time point isolates were performed using the ABySS program (47). Isolate assemblies were compared to 14 complete *S. aureus* genome sequences taken from the NCBI RefSeq database (48), along with 5 *S. aureus* clinical isolate genome sequences previously characterized by Emory Healthcare (33), using the MUMmer program (49). The all-against-all pairwise average nucleotide identities computed with MUMmer were converted to *p*-distances and used to reconstruct an *S. aureus* whole-genome phylogeny with the neighbor-joining algorithm (50) implemented in the MEGA program (51). This phylogeny was used to select the most closely related complete *S. aureus* genome sequence(s) for subsequent population genomic analyses.

### Population genomic analyses.

Individual sequence reads from each of the three time point isolates were mapped to *S. aureus* reference sequence(s) using the BWA program (52). Individual nucleotide variants (SNPs) were called on the basis of the resulting read-to-genome alignments using SAMtools (53) and GATK (54). Results from these two programs were virtually identical, and the results from SAMtools are presented here. Site-specific variant calls were used to model allelic variation within each time point isolate population. Quality control measures based on the mapping quality and read depth were used to ensure the reliability of the site-specific variant calls used to represent allelic differences. The mean BWA (Phred-like) mapping quality score was 56.4, and the mean read depth was 481. A lower bound depth cutoff of 98 reads per position (corresponding to 1.2 standard deviations from the mean) was used for calculating site-specific allelic diversity. A number of population genomic parameters were calculated on the basis of the site-specific allelic diversity values as described below.

Site-specific variant allele frequencies (VAF_*i*_) are computed as VAF_*i*_ = *v_i_*/*n_i_* where *v_i_* is the number of variant nucleotides at site *i* (i.e., nucleotides that differ from the consensus sequence) and *n_i_* is the total number of aligned nucleotides at that site. VAF_*i*_ values were used together with site-specific read depth values in order to compute the number of clones present in each of the three time point isolate populations using a kernel density method implemented in the SciClone program (37). Site-specific heterozygosity (*H_i_*) values were computed as *H_i_* $=1-\underset{\text{ATCG}}{\sum }p^{2}$ where p is the frequency of each nucleotide at site *i*.

Population genomic differentiation between time point isolates was computed using site-specific variant allele frequency differences (ΔVAF_*i*_) and polarized site-specific fixation indices (*F_STi_*). The site-specific variant allele frequency differences were calculated as follows: ΔVAF_*i*_ = *t*3VAF*_i_* − *tx*VAF*_i_* where *t*3 is time point three (VISA isolate) and *tx* can be time point one or time point two (VSSA isolate). The polarized site-specific fixation indices were calculated as follows: *F_STi_* = (*H_Ti_* − *H_Si_*)/*H_Ti_* where *H_Ti_* is the heterozygosity computed with both time points considered as a single metapopulation, and *H_Si_* is the heterozygosity computed individually for both time point isolate subpopulations. The *F_STi_* values were polarized by assigning a negative value to sites with variant allele frequencies higher at time point one or two and a positive value to sites with variant allele frequencies higher at time point three. ΔVAF and polarized *F_STi_* distributions were parameterized in order to identify statistically significant outliers using a *Z* test. A Bonferroni’s correction, based on the approximate number of sites analyzed genome-wide (2 million), was used to compute a *P* value significance threshold of 10^−8^.

### Functional enrichment analysis.

The genomic distributions of variant nucleotide sites with statistically significant time point isolate population differentiation values (for ΔVAF_*i*_, polarized *F_STi_*, or both) were evaluated with respect to their presence in a set of 24 candidate genes previously implicated in the VISA phenotype (19). To do this, a Fisher exact test was used to compare the observed number of such sites that overlap candidate genes compared to the expected number of sites computed on the basis of their genomic background density. An outlier approach was used to search for functional enrichment of novel genes not previously implicated in the VISA phenotype. To do this, genes that overlap with the top 500 most statistically significant sites for each test were analyzed. These genes were expected to identify functionally coherent gene sets (families), and enrichment values for these families were manually computed using Fisher exact test in the same way as described above.

### Protein domain architecture characterization.

Protein domain architectures for members of the LPXTG cell wall anchor domain protein family were characterized using the SMART tool with both the default SMART and Pfam domain databases (55, 56). The protein domain architecture diagrams were drawn using the MyDomains tool from ExPASy PROSITE (57).

### Ethics statement.

This study and patient confidentiality were covered under the Emory Institutional Review Board (IRB) approval (approval no. 50685). Patient isolates were collected under this study as discarded clinical samples based on the IRB approval to perform retrospective chart review and analysis.

### Accession numbers.

The raw sequence reads of the three time point isolates can be accessed from NCBI SRA (Table 1). The SRA accession numbers of the three time point isolates VSSA-T1, VSSA-T2, and VISA-T3 are SRX689719, SRX689725, and SRX689726, respectively.
